# Association between serum high-sensitivity C-reactive protein levels and migraine: a REFORM study

**DOI:** 10.1007/s10072-025-08738-y

**Published:** 2025-12-17

**Authors:** Betel Tesfay, Håkan Ashina, William Kristian Karlsson, Rune Häckert Christensen, Haidar M. Al-Khazali, Dorte Aalund Olsen, Jonna Skov Madsen, Messoud Ashina

**Affiliations:** 1 Department of Neurology, Danish Headache Center, Copenhagen University Hospital – Rigshospitalet, Copenhagen, Denmark; 2 Department of Clinical Medicine, Faculty of Health and Medical Sciences, University of Copenhagen, Copenhagen, Denmark; 3 Translational Research Center, Copenhagen University Hospital – Rigshospitalet, Copenhagen, Denmark; 4 Department of Biochemistry and Immunology, Lillebaelt Hospital, University Hospital of Southern Denmark, Vejle, Denmark; 5 Department of Regional Health Research, Faculty of Health Sciences, University of Southern Denmark, Odense, Denmark

**Keywords:** Headache, Migraine, C-reactive protein, Inflammation, Biomarker

## Abstract

**Background:**

High-sensitivity C-reactive protein (hs-CRP) is a well-established biomarker of systemic inflammation and endothelial dysfunction. Its role in capturing inflammatory processes underlying migraine remains unclear. We aimed to determine whether serum hs-CRP levels are associated with migraine.

**Methods:**

This cross-sectional study enrolled adult participants with migraine and sex-matched healthy controls (HCs). Serum hs-CRP concentrations (mg/L) were measured using a validated assay. Participants with migraine were categorized into subgroups based on type (with aura, without aura, chronic, episodic) and headache status at sampling (ictal, interictal). The primary outcome was the percentage difference in serum hs-CRP concentrations between participants with migraine and HCs. Secondary outcomes included comparisons of hs-CRP levels across migraine subgroups and between each subgroup and HCs. Multivariate regression models were used to assess associations between hs-CRP and migraine after adjusting for age, sex, body mass index, and smoking status.

**Results:**

A total of 642 participants with migraine and 154 sex-matched HCs were enrolled. Of these, 630 with migraine (565 [89.7%] females) and 153 HCs (131 [85.6%] females) provided eligible outcome data. Participants with migraine exhibited significantly higher hs-CRP concentrations than HCs, with an average increase of 31.2% (95% CI, 9.4–57.3%; *P* = 0.003). Subgroup analyses showed higher hs-CRP in migraine with aura (47.0% above HCs; *P* = 0.002) and chronic migraine (33.5% above HCs; *P* = 0.009).

**Conclusions:**

Elevated hs-CRP levels were identified in adults with migraine, implicating low-grade inflammation in migraine pathogenesis.

**Supplementary Information:**

The online version contains supplementary material available at 10.1007/s10072-025-08738-y.

## Introduction

The precise neurobiologic mechanisms underlying migraine remain elusive, yet they are believed to involve pro-inflammatory mediators [1, 2]. These mediators, including specific cytokines, chemokines, prostaglandins, and neuropeptides, promote neurogenic inflammation and meningeal nociceptor activation [1, 3–5]. Furthermore, recent advances have implicated these pro-inflammatory mediators in migraine chronification and migraine aura [2–9], potentially indicating processes occurring within the central nervous system.

C-reactive protein (CRP) is a biomarker of systemic inflammation and has been proposed as a candidate biomarker for migraine [5, 10, 11]. This pentameric protein is predominantly synthesized by hepatocytes during acute and chronic inflammation, primarily in response to interleukin-6 [12–15]. However, conventional CRP assays may lack the sensitivity needed to detect low-grade inflammation [16, 17], which could be present in migraine. To overcome this issue, high-sensitivity CRP (hs-CRP) assays provide superior precision [12, 18], enabling the detection of subtle increases in CRP concentrations.

Despite this, studies assessing the relationship between circulating hs-CRP levels and migraine have produced conflicting results [4, 19–31]. Some reports identified higher hs-CRP levels in people with migraine compared to healthy controls (HCs) [4, 23–27, 31], while others found no significant differences [19–22, 28–30]. These inconsistencies likely result from methodological variations, differences in migraine classification, small sample sizes [3, 5, 31], and inconsistent adjustment for confounders such as age, sex, body mass index (BMI), and smoking status [32–35].

To address these limitations, we conducted a large, cross-sectional investigation to compare serum hs-CRP concentrations between adults with migraine and HCs, accounting for potential confounders. In addition, we performed subgroup analyses to explore whether hs-CRP levels varied according to migraine type (with aura, without aura, chronic, episodic) and headache status at the time of blood sampling (ictal, interictal).

## Methods

The presented data derive from the parental *Re*gistry *for M*igraine (REFORM) study. A detailed description of the REFORM study has been published elsewhere [36]. All participants provided written informed consent prior to enrollment. The study protocol received approval from Scientific Ethics Committee for the Capital Region of Denmark. All procedures complied with the principles outlined in the Declaration of Helsinki, with later revisions [37]. Reporting adhered to the Strengthening the Reporting of Observational Studies in Epidemiology (STROBE) statement [38].

### Design

This cross-sectional study examined serum hs-CRP concentrations in adult participants with migraine and HCs. Enrollment spanned from October 2020 to June 2022. Participants with migraine were mainly identified from the outpatient clinic of the Danish Headache Center, Department of Neurology, Copenhagen University Hospital – Rigshospitalet. HCs were recruited using advertisement posted on a web-based recruitment platform for research volunteers (https://forsoegsperson.dk).

### Population

The full list of inclusion and exclusion criteria for participants with migraine and HCs has been published elsewhere [36]. In brief, eligible participants were adults (≥ 18 years) diagnosed with migraine without aura, migraine with aura, or chronic migraine, according to the International Classification of Headache Disorders, 3rd edition (ICHD-3) [39]. To ensure sufficient disease burden, participants with migraine were required to report experiencing an average of ≥ 4 monthly migraine days (MMDs) during the 3 months preceding enrollment. Participants were permitted to continue their preventive medication(s) if their dosing regimen had remained stable for ≥ 2 months prior to enrollment. The main exclusion criteria included a personal history of hemiplegic migraine, cluster headache, or post-traumatic headache. Additional exclusions applied to individuals with current immunosuppressive medication use, as these agents might influence serum hs-CRP concentrations [40].

For the HC group, we enrolled adults (≥ 18 years) with no personal history of any headache disorder, except for infrequent episodic tension-type headache. To further minimize potential confounders, HCs were required to have no daily medication use, except for oral contraceptives.

### Procedures

#### Clinical data assessment

Participants with migraine underwent a semi-structured interview conducted by site investigators. These interviews captured detailed sociodemographic information, headache features, comorbid conditions, and both current and past use of headache medication. In parallel, HCs underwent a more concise semi-structured interview that recorded sociodemographic data.

At the time of blood sampling, additional clinical data were recorded, including the presence and characteristics of headache, associated symptoms, and the use of acute medication within the preceding 72 h.

#### Blood sampling and analysis

Blood samples (8 mL) were obtained at baseline from an antecubital vein using intravenous cannulation and collected in serum gel-separator clot-activator tubes. Samples were maintained at room temperature for 30 min to allow complete clotting. Following clotting formation, tubes were centrifuged at 2200 × g for 10 min at 4℃. Serum aliquots (3.6 mL) were then carefully transferred into labelled cryotubes and stored at − 80 °C until analysis.

Serum hs-CRP concentrations were quantitatively determined at the Department of Biochemistry and Immunology, Lillebaelt Hospital, University of Southern Denmark. Analysis was conducted in a randomized order, blinded to subjects’ group status, to minimize potential bias. Measurements were performed using a hs-CRP assay on the Roche Cobas C702 module (Roche Diagnostics, Basel, Switzerland). This automated assay ensures robust quantification, with an analytical coefficient of variation below 9%. The lower limit of detection (LLOD) for the hs-CRP assay was 0.15 mg/L, and the functional sensitivity was 0.3 mg/L.

For hs-CRP values below the LLOD, a single imputation method was employed, replacing undetectable values with LLOD divided by 2. This approach is a widely acceptable practice to minimize bias in biomarker studies with low detection thresholds [41]. Only 3.3% of hs-CRP values were below the LLOD (17 participants with migraine, 9 HCs). For the main analysis, we chose a 20.0 mg/L cutoff for serum hs-CRP and excluded participants above this threshold [25, 42], as this cutoff has been proposed to better distinguish acute from chronic inflammation in populations with a predominantly female demographic and a high mean BMI [43]. To assess robustness, we also performed a sensitivity analysis using the conventional 10.0 mg/L cutoff [4, 29].

### Variables and outcome measures

#### Classification of participants

**Fig. 1:**
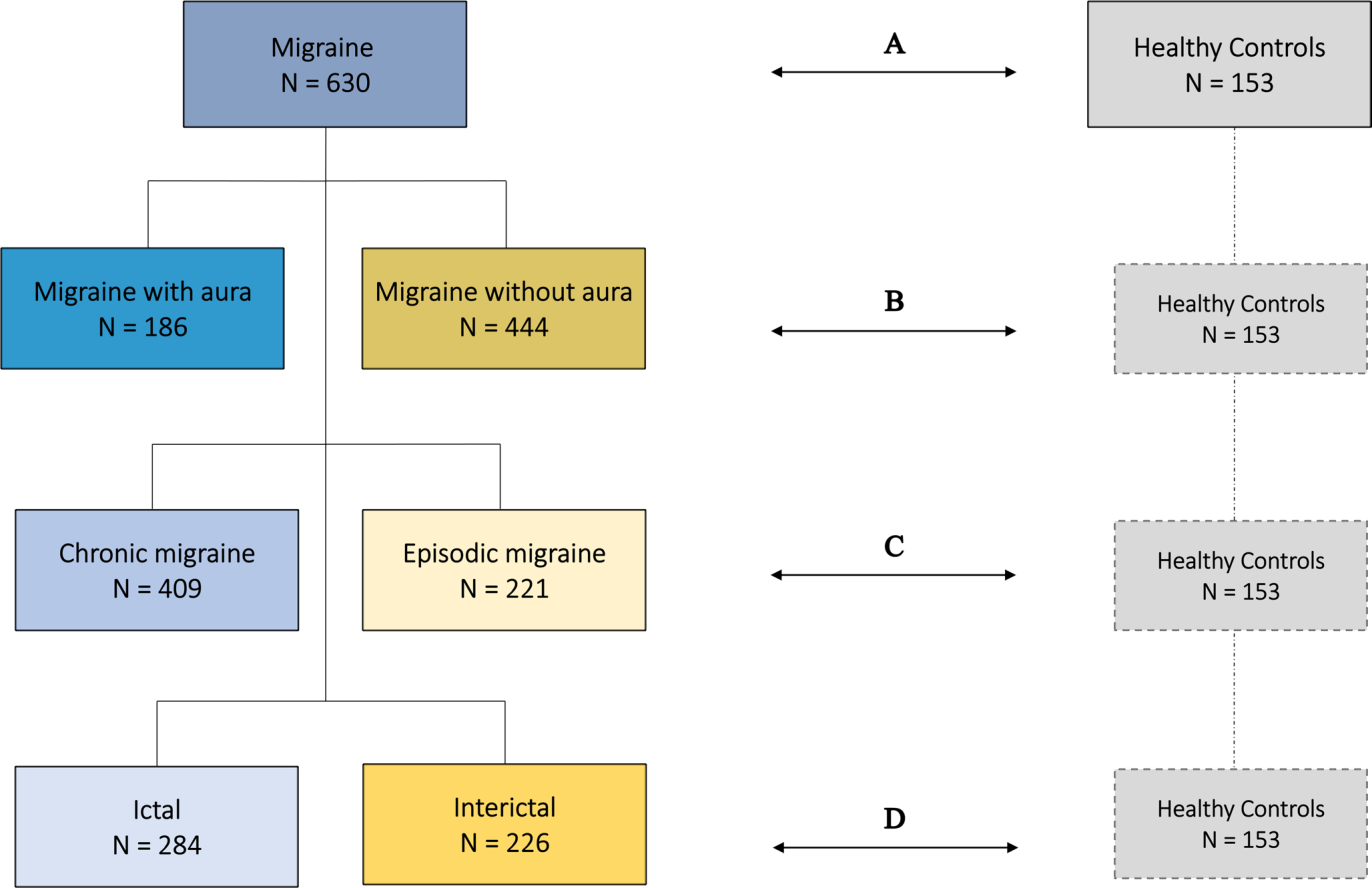
Classification of Participants with Migraine and main analysis. The participants with migraine were categorized into three main groups and compared with healthy controls. Each group was further divided into subgroups, with comparisons made within the groups and against healthy controls, as illustrated in the figure: **(A)** migraine vs. healthy controls; **(B)** migraine with aura vs. migraine without aura vs. healthy controls; **(C)** chronic migraine vs. episodic migraine vs. healthy controls; and **(D)** ictal vs. interictal vs. healthy controls

Participants with migraine were classified into three non-mutually exclusive categories according to specific clinical features (Fig. 1). Each category comprised two clearly defined subgroups to ensure comprehensive clinical differentiation. First, participants were stratified by the presence or absence of aura into either the “migraine with aura” or “migraine without aura” subgroup. Second, participants were classified based on the frequency of migraine attacks into either “chronic migraine” or “episodic migraine” subgroups. Finally, participants were categorized by their headache status at the time of blood sampling into either “ictal” or “interictal” subgroups. The ictal subgroup included participants experiencing ongoing headache and associated symptoms meeting ICHD-3 criteria for definite or probable migraine without aura. In contrast, the interictal subgroup comprised headache-free participants at the time of sampling.

#### Outcomes

An overview of the primary and secondary outcomes is presented in Fig. 1. The primary outcome was the percentage difference in serum hs-CRP concentrations between participants with migraine and HCs. The secondary outcomes included the analysis of hs-CRP concentrations across migraine subgroups and comparisons between each subgroup and HCs.

Exploratory analyses were performed to assess relationships between hs-CRP concentrations and important clinical variables. Specifically, associations were explored between serum hs-CRP levels and: (I) mean monthly headache days (MHDs); (II) mean MMDs; (III) mean MMDs with both aura and headache; (IV) mean monthly days of acute headache medication use. Clinical variables were calculated as the average number of days self-reported by participants during the month preceding enrollment.

#### Potential confounders

A comprehensive literature review identified several potential confounders that might influence the association between migraine and hs-CRP concentrations. These variables comprised demographic characteristics, clinical parameters, medication use, and comorbidities [32–35].

Demographic variables included age (continuous variable) and sex (categorized as female or male) [32, 33]. Clinical parameters encompassed BMI (continuous variable) and smoking status (current vs. non-smoker) [34, 35]. Medication use was also systematically recorded to account for its potential impact on hs-CRP concentrations [44–46]. This included medication-overuse (ongoing vs. absent), use of preventive migraine medications (current vs. not used), and statin therapy (current vs. not used). In addition, recent intake (within 72 h) of non-steroidal anti-inflammatory drugs (NSAIDs) or triptans (yes vs. no) was documented.

To account for broader inflammatory influences, psychiatric and somatic comorbidities were assessed (ongoing vs. absent). These encompassed anxiety, depression, asthma, autoimmune disorders, chronic daily low back pain (≥ 3 months), chronic daily neck pain (≥ 3 months), hypertension, other cardiovascular disorders, and past history of cancer [47–54]. Each condition was considered due to its potential association with systemic inflammation and hs-CRP variability.

### Statistical analysis

An overview of the statistical analyses is provided in eTable 1. Sampling was convenience-based, targeting enrollment of at least 600 participants with migraine and 150 HCs. Categorical variables were presented as counts and percentages, while continuous variables were reported as means with standard deviations (SDs) or medians with interquartile ranges (IQRs), depending on the data distribution. Assessments of data distributions were performed via visual inspection of histogram and quartile-quartile plots. However, *per* standard migraine research practices, mean values were reported for average values of MHDs, MMDs, MMDs with both aura and headache, and monthly days of acute headache medication use. Baseline characteristic comparisons between participants with migraine and HCs were conducted using unpaired t-tests for continuous variables and Pearsons ꭓ^2^-test for categorical variables.

Differences in serum hs-CRP concentrations between participants and HCs, as well as comparisons within migraine subgroups, were examined using linear regression. The following models were applied:

I. Unadjusted analysis (hs-CRP as the dependent variable).
II. Adjusted model controlling for age, sex, BMI, and smoking status.
III. Fully adjusted multivariate model including all potential confounders and clinical migraine characteristics (chronic migraine, migraine with aura, ictal status).

Given the skewed distribution of serum hs-CRP residuals, logarithmic transformation was applied to approximate normalization of the data before analysis. Accordingly, results from the linear regression models were expressed as percentage differences, with estimates back-transformed alongside 95% confidence intervals (CIs). Plots were generated using logarithmically transformed y-axes. All regression models underwent diagnostic evaluation, including assessment of multicollinearity, with a variance inflation factor threshold of < 5.

Spearman rank correlation analyses were performed to explore relationships between serum hs-CRP concentrations and important clinical features, including:

I. Mean MHDs.
II. Mean MMDs.
III. Mean MMDs with both aura and headache.
IV. Mean monthly days of acute headache medication use.

The proportion of missing data for covariates was < 5%, except for recent NSAIDs and triptan use, which were included only in the fully adjusted model. The primary analysis was conducted using a complete case approach. To assess the impact of missing covariate data, a sensitivity analysis was performed using multiple imputation by chained equations, generating 20 imputed datasets.

All statistical tests were two-sided, with a *P* value < 0.05 considered statistically significant. The Bonferroni correction was applied to adjust for multiple comparisons within each sub-analysis cluster, resulting in three pairwise comparisons (e.g., migraine without aura vs. HC, migraine with aura vs. HCs, migraine without aura vs. migraine with aura). All analysis were conducted using R statistical software (version 4.3.3).

## Results

A total of 642 participants with migraine and 154 HCs were enrolled. Of these, seven participants with migraine were excluded due to concurrent use of immunosuppressive medication. In addition, five participants with migraine and one HC were excluded because their serum hs-CRP concentrations exceeded the upper cutoff of 20.0 mg/L. The final analytical sample comprised 630 participants with migraine and 153 HCs.

Baseline demographic and clinical characteristics are summarized in Table 1 and eTable 2, with additional details on headache status, recent acute treatment, and treatment history presented in eTable 3 and eTable 4. Participants with migraine had a mean age of 44.1 (SD, 12.2), and most were female (*n* = 565, 89.7%). Among participants with migraine, 186 (29.5%) had migraine with aura, and 409 (64.9%) met the criteria for chronic migraine.

**Table 1:** Clinical and sociodemographic characteristics

Demographics	Migraine	Healthy controls	Chronic migraine	Episodic Migraine	Migraine without aura	Migraine with aura
Participants, No.	630	153	409	221	444	186
Age, mean (SD), y	44.1 (12.2)	41.3 (11.8)	43.4 (12.2)	45.5 (12.0)	43.9 (12.3)	44.6 (12.0)
Female sex, No. (%)	565 (89.7%)	131 (85.6%)	371 (90.7%)	194 (87.8%)	396 (89.2%)	169 (90.9%)
Body mass index, mean (SD), kg/m2	25.1 (4.9)	24.7 (4.0)	25.4 (5.2)	24.6 (4.3)	25.0 (4.7)	25.4 (5.4)
Current smokers,^a^ No. (%)	65 (10.5%)	20 (13.5%)	43 (10.7%)	22 (10.1%)	49 (11.1%)	16 (8.7%)
**Clinical characteristics**						
Migraine with aura, No. (%)	186 (29.5%)	-	131 (32.0%)	65 (24.9%)	-	186 (100%)
Migraine and aura frequency,^b^ mean (SD)						
MHDs	19.7 (7.8)	-	23.3 (6.1)	13.0 (5.9)	19.6 (8.0)	19.9 (7.5)
MMDs	14.4 (6.9)	-	17.3 (6.7)	9.2 (3.3)	14.3 (6.9)	14.8 (7.1)
MMDs with both headache and aura	4.0 (5.1)	-	4.8 (5.8)	2.0 (2.7)	-	4.0 (5.2)
Comorbidities, No. (%)						
Asthma	66 (10.5%)	-	48 (11.7%)	18 (8.1%)	39 (8.8%)	27 (14.5%)
Autoimmune disorders	69 (10.8%)	-	47 (11.5%)	21 (9.5%)	48 (10.8%)	20 (10.8%)
Daily neck pain	95 (15.1%)		82 (20.0%)	13 (5.9%)	68 (15.3%)	27 (14.5%)
Daily low back pain	60 (9.5%)		47 (11.5%)	13 (5.9%)	46 (10.4%)	14 (7.5%)
History of cancer^c^	32 (5.1%)	-	28 (6.9%)	4 (1.8%)	22 (5.0%)	10 (5.4%)
Hypertension	68 (10.8%)	-	52 (12.7%)	16 (7.2%)	54 (12.2%)	14 (7.5%)
Other cardiovascular disorders	39 (6.2%)	-	29 (7.1%)	10 (4.5%)	25 (5.6%)	14 (7.5%)
Anxiety	63 (10.0%)	-	48 (11.7%)	15 (6.8%)	43 (9.7%)	20 (10.8%)
Depression	63 (10.0%)	-	42 (10.3%)	21 (9.5%)	43 (9.7%)	20 (10.8%)

Abbreviations: MHDs, monthly headache days; MMDs, monthly migraine days; SD, standard deviation.

Symbols: ^a^, Data missing in 9 participants with migraine (six with chronic migraine, three with episodic migraine, five with migraine *with* aura, and four with migraine *without* aura) and five healthy controls; ^b^, mean over a 1-month period prior to study enrolment; -, not applicable; ^c^, without current cancer diagnosis

**Table 2:** Hs-CRP concentrations in participants with migraine, migraine Subgroups, and healthy controls

Serum hs-CRP concentration (mg/L)			
**Total population**	**No.**	**Mean (SD)**	**Median (IQR)**
All participants with migraine	630	1.84 (2.79) *	0.68 (0.39–1.98) *
Healthy controls	153	1.43 (2.52)	0.54 (0.29–1.11)
**History of aura**			
Migraine with aura	186	2.14 (3.16) *	0.73 (0.41–2.40) *
Migraine without aura	444	1.71 (2.61)	0.67 (0.38–1.86)
**Diagnosis of chronic migraine**			
Chronic migraine	409	1.93 (2.80) *	0.76 (0.39–2.18) *
Episodic migraine	221	1.67 (2.78)	0.60 (0.38–1.53)
**Headache status at blood sampling**			
Ictal	284	1.86 (2.75) *	0.73 (0.39–1.96) *
Interictal	226	1.99 (3.04) *	0.65 (0.40–2.31) *

Abbreviations: IQR, interquartile range; SD, standard deviation, Hs-CRP, high-sensitivity C- reactive protein.

Symbols: *, Statistically significant differences (*P* values < 0.05) in linear models compared with healthy controls

A total of 346 (54.9%) participants reported medication-overuse and 316 participants (50.2%) had concurrent use of preventive migraine medication (eTable 3). Data regarding intake of acute medication within the 72 h preceding blood sampling was available from 577 participants with migraine. Among these, 166 (28.8%) participants reported an intake of NSAIDs, while 205 (35.5%) reported an intake of triptans (eTable 3).

HCs had a comparable proportion of females (*n* = 131, 85.6%; *P* = 0.15). However, they were slightly younger than participants with migraine (mean age, 41.3 years; SD, 11.8; *P* = 0.006). No significant differences were observed in BMI (*P* = 0.70) or smoking status (*P* = 0.31) between the two groups.

Information on headache status at the time of blood sampling was available for 626 participants with migraine. Among these, 284 (45.4%) participants with migraine were classified as “ictal”. Of these, 161 (25.7%) met the ICHD-3 criteria for definite migraine without aura, whilst 123 (19.6%) fulfilled the criteria for probable migraine. An additional 116 participants (18.5%) reported non-migraine headache, whereas 226 (36.1%) were classified as interictal at the time of sampling (eTable 3).

### Migraine and migraine subgroups

**Fig. 2:**
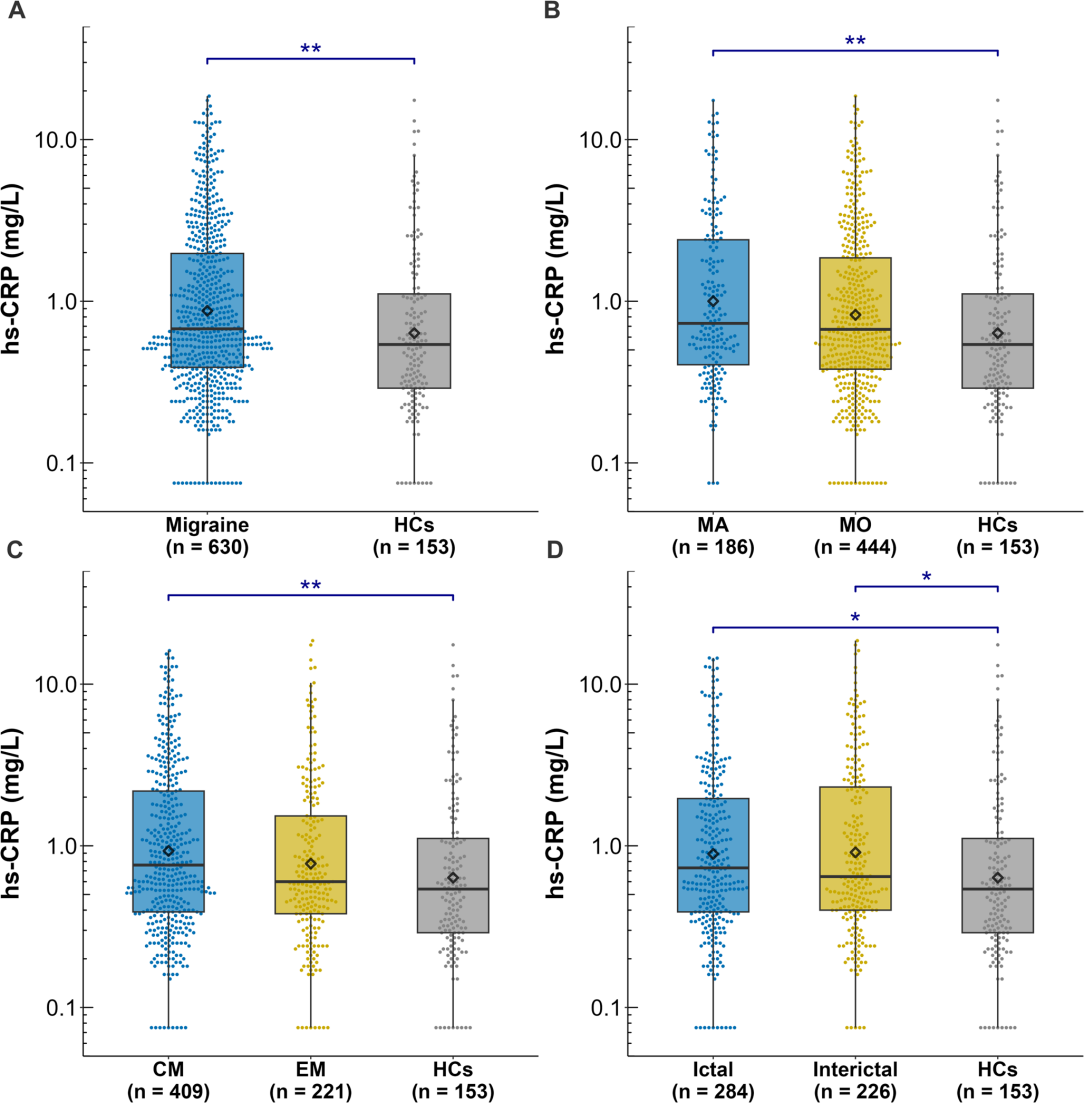
Serum hs-CRP concentrations in primary and secondary outcomes. Serum hs-CRP concentrations plotted on a logarithmic scale in **(A)** Participants with migraine and healthy controls (HCs); **(B)** migraine with aura (MA) group, migraine without aura (MO) group and HCs; **(C)** chronic migraine (CM) group, episodic migraine (EM) group, and HCs; and **(D)** ictal group, interictal group and HCs. Box plots depict median (horizontal bar), IQR (hinges), and 1.5 × IQR (whiskers). *P values < 0.05. **P values < 0.01

Serum hs-CRP concentrations, reported as mean (SD) and median (IQR), for the overall migraine population, its subgroups, and HCs, are summarized in Table 2 and visually represented in Fig. 2. All *P* values in this section were adjusted for age, sex, BMI, and smoking status. Additional analyses adjusting for the full set of confounders yielded similar findings, with estimated relative differences and 95% CIs provided in eTable 5.

Participants with migraine exhibited significantly higher serum hs-CRP concentrations compared to HCs, with an average increase of 31.2% (95% CI, 9.4–57.3%; *P* = 0.003). The subgroup analyses revealed that participants with migraine with aura also had high serum hs-CRP concentrations, which were 47.0% greater than HCs (95% CI, 12.3–92.3%; *P* = 0.002). Likewise, participants with chronic migraine exhibited hs-CRP concentrations 33.5% higher than HCs (95% CI, 5.7–68.6%; *P* = 0.009). No significant differences in hs-CRP concentrations were found between participants with migraine without aura and HCs, migraine with aura and migraine without aura, episodic migraine and HCs, or chronic migraine and episodic migraine (all *P* values ≥ 0.05).

### Ictal vs. Interictal status

When assessing headache status at the time of blood sampling, both ictal and interictal participants with migraine exhibited significantly higher hs-CRP concentrations compared with HCs (eTable 5; Fig. 2D).

### Correlation analyses

A weak positive correlation was identified between serum hs-CRP concentrations and mean MHDs (*n* = 630; *r*_*s*_ = 0.083; *P* = 0.038). However, no significant correlations were observed between hs-CRP concentrations and mean MMDs (*n* = 630; rₛ = 0.036; *P* = 0.36), mean MMDs with both aura and headache (*n* = 186; rₛ = 0.12; *P* = 0.11), or mean monthly days of acute headache medication use (*n* = 628; rₛ = 0.07; *P* = 0.07).

### Sensitivity analyses

Sensitivity analyses were conducted to assess the robustness of our findings. Results from multiple imputation of missing data were consistent with the primary analyses (eTable 6). Further analyses excluding serum hs-CRP values above 10.0 mg/L also supported the main results (eTable 7). These analyses revealed additional significant differences between groups. Participants with migraine without aura exhibited 26.3% higher serum hs-CRP concentrations, compared with HCs (95% CI: 1.6%–57.0%; *P* = 0.031).

## Discussion

This cross-sectional study provides compelling evidence that serum hs-CRP concentrations are elevated in adults with migraine, particularly in those with migraine with aura and chronic migraine, compared with HCs. The identified associations remained significant after adjusting for age, sex, BMI, and smoking status, underscoring that elevated hs-CRP levels are independently related to migraine. Collectively, our results implicate low-grade systemic inflammation and possibly also endothelial dysfunction in migraine pathophysiology. Furthermore, persistent inflammatory activity might contribute to disease progression and headache chronification, a hypothesis supported by the weak but statistically significant association between hs-CRP levels and MHDs. However, the very weak correlation indicates that MHDs as a standalone factor is of limited clinical relevance.

### Existing evidence and added value

Numerous previous studies have examined the relationship between circulating CRP concentrations and migraine, but results have been incongruent [4, 19–31]. Some reports found higher hs-CRP levels in people with migraine [4, 23–27, 31], with some specifically implicating migraine with aura [24, 25, 31], while other studies observed no difference compared to HCs [19–22, 28–30]. A review of 12 studies and a meta-analysis of six case-control studies suggests an association between elevated blood CRP or hs-CRP concentrations and migraine [3, 5]. However, the meta-analysis selectively included studies showing elevated levels in migraine participants [27, 31, 55–58], while excluding relevant studies for unspecified reasons [19–22, 28–30, 59–62]. Together, these inconsistencies underscore the impact of methodologic differences, small sample sizes, and the use of less sensitive CRP assays. In light of these limitations, our investigation incorporated a large, well-characterized sample, used standardized hs-CRP assays, and adjusted for potential confounders. This approach offers more definitive insights into the CRP-migraine relationship. Moreover, by capturing both ictal and interictal migraine phases, our results highlight persistent low-grade inflammation as a disease feature rather than a transient phenomenon.

### Migraine, Low-Grade systemic Inflammation, and endothelial dysfunction

Several pathophysiologic mechanisms might explain elevated hs-CRP levels in migraine. First, neurogenic inflammation—initiated by trigeminal sensory neuron activation—triggers the release of vasoactive neuropeptides (e.g., calcitonin gene-related peptide, substance P) [2, 6, 63, 64]. These neuropeptides mediate dural vasodilation, mast cell degranulation, and modulate nociceptive transmission [6, 64, 65]. This cascade of local inflammatory events might ultimately propagate systemic immune signaling, resulting in the production of pro-inflammatory cytokines (e.g., interleukin-1β, interleukin-6, and tumor necrosis factor-alpha [66, 67], which are potent inducers of CRP synthesis in human hepatocytes [68]. Second, endothelial dysfunction might concurrently increase hs-CRP concentrations. Migraine with aura has been repeatedly associated with endothelial dysfunction and increased risk of vascular events [69–73], both of which are linked to elevated circulating CRP levels [74–76]. These systemic processes might interact with neurogenic inflammation in the meninges, amplifying the pathophysiologic mechanisms that underlie migraine.

### Migraine with aura: evidence of distinct inflammatory and vascular contributions

Our subgroup analysis revealed that participants with migraine with aura exhibited higher hs-CRP concentrations than HCs. However, hs-CRP levels did not differ between those with and without aura. This finding suggests that the observed association between hs-CRP and migraine with aura should be interpreted with caution. Nevertheless, mechanisms specific to aura might still contribute to low-grade systemic inflammation.

One proposed mechanism is cortical spreading depolarization (CSD), a self-propagating wave of neuronal and glial depolarization across the cerebral cortex [64, 77]. CSD alters ionic gradient, disrupts neurotransmitter homeostasis, and generates a surge of excitatory signals [78, 79]. These changes activate astrocytes, microglia, and meningeal immune cells [80, 81]. The ensuing release of cytokines and inflammatory mediators might promote sustained inflammatory processes.

This pathophysiologic cascade could plausibly extend beyond the brain, influencing peripheral immune response. Indeed, we recently identified elevated concentrations of soluble urokinase plasminogen activator receptor (suPAR) in the same participants with migraine with aura [82]. SuPAR is a marker of low-grade chronic inflammation and is associated with endothelial dysfunction and long-term cardiovascular risk [83–85].

Although a causal relationship remains unproven, our findings suggest that migraine with aura is associated with a distinct pro-inflammatory state. This state could involve heightened endothelial reactivity and systemic immune activation. The convergence of cortical, vascular, and pro-inflammatory processes might help explain observed associations between migraine with aura and increased risk of cerebro- and cardiovascular events.

### Chronic migraine: persistent Low-Grade inflammation in headache chronification

Our findings also demonstrated higher hs-CRP concentrations in chronic migraine, compared with HCs, whereas no difference was found between episodic migraine and controls. This pattern aligns with the populations-based HUNT3 study and the Tromsø study, both of which linked more frequent headache (≥ 7 days/month) to stronger inflammatory signatures [25, 42]. Chronic migraine is characterized by increased neuronal excitability and central sensitization —physiologic changes that might be exacerbated by repeated inflammatory stimuli [86, 87]. Although the correlation between hs-CRP and MHDs was modest, it reinforces the possibility that persistent, low-grade inflammation contributes to disease progression and the transition from episodic to chronic migraine.

### Interictal Low-Grade inflammation: evidence for a persistent trait

An important finding in our study is that both ictal and interictal participants with migraine exhibited higher hs-CRP concentrations compared with HCs, whereas no significant difference was observed between the two phases. This suggests that systemic inflammation might persist independently of acute migraine attacks.

Previous studies assessing CRP or hs-CRP in migraine have not consistently accounted for headache status at the time of blood sampling. Most investigations have focused exclusively on interictal measurements [4, 21, 24, 26–28, 30, 31, 56, 60, 62, 88], with seven reporting elevated hs-CRP levels in participants with migraine relative to controls [4, 24, 26, 27, 31, 56, 88]. However, studies comparing ictal and interictal CRP concentrations are few and inconclusive [22, 58, 61]. Two found no difference between the phases [22, 61], while a more recent investigation reported higher hs-CRP levels during the ictal phase [58].

Taken together, these conflicting findings underscore a lack of consensus in the literature regarding the temporal dynamics of inflammation in migraine. Our results lend support to the emerging view that inflammation is not merely a reactive phenomenon limited to acute attacks but could instead represent a persistent trait of the disorder. This chronic pro-inflammatory state might involve sustained immune activation and contribute to underlying endothelial dysfunction. However, our interictal participants might have experienced a migraine attack shortly before or after blood sampling. Thus, subclinical fluctuations in inflammatory markers cannot be entirely excluded. In addition, the sample size was not sufficient to examine the timing of attack onset in finer detail.

Despite these caveats, our findings challenge the traditional paradigm of migraine as an episodic inflammatory disorder and instead support the hypothesis that systemic inflammation might be a persistent feature of the disease.

### Implications for migraine pathophysiology and future research

Our findings have critical implications for understanding migraine as a disorder with inflammatory and vascular components. First, the association between migraine with aura, hs-CRP elevation, and endothelial dysfunction supports the hypothesis that vascular mechanisms play a key role in migraine pathogenesis. Second, the elevation of hs-CRP in chronic migraine suggests that low-grade systemic inflammation might contribute to migraine progression and central sensitization. Lastly, the observation that hs-CRP remains elevated in the interictal phase indicates that inflammation in migraine is not merely a byproduct of acute attacks but reflects a persistent disease trait.

Future studies should investigate whether hs-CRP fluctuations correlate with migraine onset, attack severity, or response to treatment. In addition, longitudinal studies are needed to determine whether persistent inflammation precedes migraine onset or is a consequence of repeated attacks. Interventional trials assessing whether anti-inflammatory or endothelial-targeted therapies can modulate hs-CRP levels and improve migraine outcomes could offer new therapeutic insights.

### Limitations

This study has several limitations. Its cross-sectional design precludes drawing causal inferences between hs-CRP concentrations and migraine. Although we adjusted for multiple confounding factors, residual confounding from factors such as physical activity, insomnia, and diet may persist [25, 89–92]. The wide variation in the measurements suggests that our sample size was insufficient to detect differences in specific subgroups, such as between migraine with and without aura or episodic versus chronic migraine. Additionally, there was an age mismatch between participants with migraine and HCs, which we adjusted for in our statistical analyses. With regard to infections, participants who reported overt symptoms were rescheduled to a separate day, and those with hs-CRP levels above predefined thresholds were excluded to limit the influence of acute inflammation. Nevertheless, we cannot fully exclude the possibility that subclinical infections affected hs-CRP concentrations. Finally, our participants were primarily recruited from a tertiary headache clinic and were predominantly categorized as white, which may introduce sampling bias and limit the generalizability of our findings to broader migraine population.

## Conclusions

Our study, involving a large cohort of participants, identified elevated serum hs-CRP concentrations in participants with migraine. Notably, this elevation was not limited to the immediate presence of migraine headache, indicating a persisting inflammatory response. Our findings underscore the importance of future prospective studies to further elucidate the clinical relevance of serum hs-CRP as a potential biomarker for disease activity and treatment response in migraine.

## Supplementary Information

Below is the link to the electronic supplementary material.

Supplementary Material 1 (DOCX 67.4 KB)

## Data Availability

The data analyzed in this study are available from the corresponding author upon reasonable request.

## Abbreviations

BMI: Body mass index
CI: Confidence interval
CRP: C-reactive protein
CSD: Cortical spreading depolarization
HCs: Healthy Controls
Hs-CRP: High-sensitivity C-reactive protein
ICHD-3: International Classification of Headache Disorders, 3rd edition
IQR: Interquartile range
LLOD: Lower limit of detection
MHDs: Monthly headache days
MMDs: Monthly migraine days
NSAIDs: non-steroidal anti-inflammatory drugs
REFORM: Registry for Migraine
SD: Standard deviation
STROBE: Strengthening the Reporting of Observational Studies in Epidemiology
suPAR: Soluble urokinase plasminogen activator receptor
