# Supplementary material for: Association between serum high-sensitivity C-reactive protein levels and migraine: a REFORM study
Source: Neurol Sci. 2025 Dec 17;47(1):9. doi: 10.1007/s10072-025-08738-y (PMC12708735; doi:10.1007/s10072-025-08738-y)
Supplement: Supplementary file 1 — Supplementary Material 1 (DOCX 67.4 KB) [file 10072_2025_8738_MOESM1_ESM.docx]

**Supplementary Material**

**Title:** Association Between Serum High-Sensitivity C-Reactive Protein Levels and Migraine: a REFORM Study

**Journal**: Neurological Sciences

**Authors****:** Betel *Tesfay*, MD; Håkan *Ashina*, MD, PhD; William Kristian *Karlsson*, MD; Rune Häckert *Christensen*, MD; Haidar M. *Al-Khazali*, MD; Dorte Aalund *Olsen*, MSc; Jonna Skov *Madsen*, MD, PhD; Messoud *Ashina*, MD, PhD, DMSci

**Corresponding Author:** Messoud Ashina, MD, PhD, DMSc

Department of Neurology, Danish Headache Center, Copenhagen University Hospital – Rigshospitalet, Copenhagen, Denmark.

Email: [ashina@dadlnet.dk](mailto:ashina@dadlnet.dk)

Table of Contents

[eTable 1. Overview of statistical analyses 2](#_Toc1597307030)

[eTable 2. Demographics and clinical characteristics according to headache status 3](#_Toc1298517438)

[eTable 3. Headache status and treatment history according to migraine sugbroups 4](#_Toc925767519)

[eTable 4. Acute treatments and treatment history according to headache status 5](#_Toc676754505)

[eTable 5. Estimated relative differences in serum hs-CRP 6](#_Toc207597601)

[eTable 6. Estimated relative differences in serum hs-CRP (sensitivity analysis; imputed) 7](#_Toc2101645191)

[eTable 7. Estimated relative differences in serum hs-CRP (sensitivity analysis; exclusion of hs-CRP values >10.0 mg/L) 8](#_Toc1519623064)

# **eTable 1.** Overview of statistical analyses

| **Comparison** | **Method of analysis** |
| --- | --- |
| Data distribution | Histogram and QQ-plots |
| Comparison of serum hs-CRP concentrations | Linear regression models: 1) unadjusted.  2) adjusted for age, sex, BMI, and smoking status.  3) fully adjusted, including potential confounders: age, sex, body mass index, smoking status, chronic migraine, migraine with aura, ictal status, medication-overuse, use of preventive medications, use of statins, intake of NSAIDs within 72 hours, intake of triptans within 72 hours, anxiety, depression, asthma, autoimmune diseases, daily low back pain, daily neck pain, hypertension, and cardiovascular diseases. |
| Correlation between serum hs-CRP concentrations and (1) mean monthly headache days, (2) mean monthly migraine days, (3) mean monthly migraine days with both aura and headache, and 4) mean monthly days with acute medication use. | Spearman’s rank correlation coefficient |
| Multiple hypothesis testing adjustment | Bonferroni method |
| **Abbreviations**: BMI, Body mass index; hs-CRP, high-sensitivity C-reactive protein; NSAIDs, non-steroidal anti-inflammatory drugs; QQ-plots, quantile-quantile plots. | |

eTable 2. Demographics and clinical characteristics according to headache status

| **Demographics** | **Ictal** | **Interictal** |
| --- | --- | --- |
| **Participants**, No. | 284 | 226 |
| **Age**, mean (SD), y | 43.4 (12.2) | 45.0 (11.4) |
| **Female sex**, No. (%) | 258 (90.8%) | 207 (91.6%) |
| **Body mass index**, mean (SD), kg/m^2^ | 25.3 (5.0) | 25.1 (4.9) |
| **Current smokers**,^a^ No. (%) | 33 (11.8%) | 19 (8.5%) |
| **Clinical characteristics** |  |  |
| Chronic migraine, No. (%) | 215 (75.7%) | 104 (46.0%) |
| Migraine with aura, No. (%) | 86 (30.3%) | 67 (29.6%) |
| **Migraine and aura frequency**,^b^ mean (SD) |  |  |
| MHDs | 21.9 (7.5) | 15.4 (6.5) |
| MMDs | 16.8 (7.4) | 12.0 (5.5) |
| MMDs with both headache and aura | 4.6 (5.4) | 3.6 (5.6) |
| **Comorbidities,** No. (%) |  |  |
| Asthma | 36 (12.7%) | 17 (7.5%) |
| Autoimmune diseases | 30 (10.6%) | 25 (11.1%) |
| History of cancer^c^ | 15 (5.3%) | 9 (4.0%) |
| Daily neck pain | 54 (19.0%) | 20 (8.8%) |
| Daily low back pain | 34 (12.0%) | 8 (6.9%) |
| Hypertension | 29 (10.2%) | 24 (10.6%) |
| Other cardiovascular disorders | 13 (4.6%) | 15 (6.6%) |
| Anxiety | 33 (11.6%) | 22 (9.7%) |
| Depression | 38 (13.4%) | 17 (7.5%) |
| **Abbreviations:** MHDs, monthly headache days; MMDs, monthly migraine days; SD, standard deviation.  **Symbols:** -, not applicable; ^a^, data missing in four participants with ictal status and two participants with interictal status; ^b^, mean over a 1-month period prior to study enrolment; ^c^, without current cancer diagnosis. | | |

eTable 3. Headache status and treatment history according to migraine subgroups

| **Status at blood sampling** | **Migraine** | **Chronic migraine** | **Episodic migraine** | **Migraine with aura** | **Migraine without aura** |
| --- | --- | --- | --- | --- | --- |
| **Participants with data on headache**, No. | 626 | 406 | 220 | 185 | 441 |
| Ictal | 284 (45.4%) | 215 (53.0%) | 69 (31.4%) | 86 (46.5%) | 198 (44.9%) |
| Definite migraine | 161 (25.7%) | 126 (31.0%) | 35 (15.9%) | 49 (26.5%) | 112 (25.4%) |
| Probable migraine | 123 (19.6%) | 89 (21.9%) | 34 (15.5%) | 37 (20.0%) | 86 (19.5%) |
| Interictal | 226 (36.1%) | 104 (25.6%) | 122 (55.5%) | 67 (36.2%) | 159 (36.1%) |
| **Recent acute medication intake**,^a^ No. (%) |  |  |  |  |  |
| NSAIDs | 166 (28.8%) | 125 (33.1%) | 41 (20.6%) | 45 (26.3%) | 121 (29.8%) |
| Triptans | 205 (35.5%) | 138 (36.5%) | 67 (33.8%) | 48 (28.1%) | 157 (38.8%) |
| Missing data | 53 | 31 | 22 | 15 | 38 |
| **Treatment history** |  |  |  |  |  |
| Participants with data on treatment history, No. | 630 | 409 | 221 | 186 | 444 |
| Medication-overuse, No. (%) | 346 (54.9%) | 241 (58.9%) | 105 (47.5%) | 96 (51.6%) | 250 (56.3%) |
| Concurrent preventive migraine medication, No. (%) | 316 (50.2%) | 193 (47.2%) | 123 (55.7%) | 91 (48.9%) | 225 (50.7%) |
| Concurrent use of statins, No. (%) | 27 (4.3%) | 17 (4.2%) | 10 (4.5%) | 11 (5.9%) | 16 (3.6%) |
| Monthly days with use of acute medications, Mean (SD) | 11.8 (6.5) | 12.7 (6.9) | 10.2 (5.3) | 11.6 (6.7) | 11.9 (6.4) |
| **Abbreviations:** NSAIDs, non-steroidal anti-inflammatory drugs.  **Symbols:** ^a^, intake ≤ 72 h prior to blood sampling. | | | | | |

eTable 4. Acute treatments and treatment history according to headache status

| **Status at blood sampling** | **Ictal** | **Interictal** |
| --- | --- | --- |
| **Participants**, No. | 284 | 226 |
| **Recent acute medication intake**,^a^ No. (%) |  |  |
| NSAIDs | 80 (29.3%) | 51 (25.8%) |
| Triptans | 97 (35.5%) | 71 (35.9%) |
| Missing data | 11 | 28 |
| **Treatment history** |  |  |
| Medication-overuse, No. (%) | 152 (53.5%) | 57 (49.1%) |
| Concurrent preventive migraine medication, No. (%) | 137 (48.2%) | 114 (50.4%) |
| Concurrent use of statins, No. (%) | 14 (4.9%) | 7 (3.1%) |
| Monthly days with use of acute medications, Mean (SD) | 11.7 (6.7) | 12.1 (6.0) |
| **Abbreviations:** NSAIDs, non-steroidal anti-inflammatory drugs.  **Symbols:** ^a^, Intake ≤ 72 h prior to blood sampling. | | |

eTable 5. Estimated relative differences in serum hs-CRP (main analysis)

|  | **Estimated relative difference** | | | | | |
| --- | --- | --- | --- | --- | --- | --- |
|  | **Unadjusted model** | | **Adjusted for age, sex, BMI, and smoking** | | **Fully adjusted model** | |
|  | **% (95% CI)** | ***p*-value**^*^ | **% (95% CI)** | ***p*-value**^*^ | **% (95% CI)** | ***p*-value**^*^ |
| **Total population** |  |  |  |  |  |  |
| Migraine, No.; HCs, No. | 630; 153 | - | 621; 148 | - | - | - |
| Migraine vs HCs | 37.3 (11.5 to 69.2) | **0.003** | 31.2 (9.4 to 57.3) | **0.003** | - | - |
| **History of aura** |  |  |  |  |  |  |
| No. migraine; No. HCs | 630; 153 | - | 621; 148 | - | 562;0 | - |
| MA vs MO | 21.5 (-5.0 to 55.4) | 0.17 | 17.3 (-5.2 to 45.1) | 0.22 | 20.1 (-0.6 to 45.0) | 0.057 |
| MA vs HCs | 57.6 (15.8 to 114.3) | **0.001** | 47.0 (12.3 to 92.3) | **0.002** | - | - |
| MO vs HCs | 29.7 (-0.4 to 68.9) | 0.056 | 25.3 (-0.4 to 57.7) | 0.056 | - | - |
| **Diagnosis of chronic migraine** |  |  |  |  |  |  |
| No. migraine; No. HCs | 630;153 | - | 621; 148 | - | 562;0 | - |
| CM vs EM | 19.9 (-5.3 to 51.7) | 0.20 | 5.2 (-14.3 to 29.0) | > 0.99 | 1.2 (-16.6 to 22.9) | 0.90 |
| CM vs HCs | 46.4 (12.0 to 91.2) | **0.002** | 33.5 (5.7 to 68.6) | **0.009** | - | - |
| EM vs HCs | 22.1 (-9.2 to 64.3) | 0.32 | 26.9 (-2.0 to 64.4) | 0.082 | - | - |
| **Headache status at blood sampling** |  |  |  |  |  |  |
| Migraine, No.; HCs, No. | 510;153 | - | 504;148 | - | 461;0 | - |
| Ictal vs Interictal | -2.2 (-24.2 to 26.3) | > 0.99 | -4.8 (-23.6 to 18.5) | > 0.99 | -15.9 (-31.4 to 3.1) | 0.096 |
| Ictal vs HCs | 40.3 (5.2 to 87.0) | **0.015** | 31.1 (2.2 to 68.3) | **0.028** | - | - |
| Interictal vs HCs | 43.4 (6.2 to 93.5) | **0.012** | 37.8 (6.1 to 78.9) | **0.010** | - | - |
| **Abbreviations:** BMI, body mass index; CI, confidence interval; CM, chronic migraine; EM, episodic migraine; HCs, healthy controls; IQR, interquartile range; MA, migraine with aura; MO, migraine without aura; SD, standard deviation. **Symbols:** *, adjusted for multiple testing using the Bonferroni method; -, not applicable. | | | | | | |

eTable 6. Estimated relative differences in serum hs-CRP (sensitivity analysis; imputed)

|  | **Estimated relative difference** | | | | | |
| --- | --- | --- | --- | --- | --- | --- |
|  | **Unadjusted model** | | **Adjusted for age, sex, BMI, and smoking** | | **Fully adjusted model** | |
|  | **% (95% CI)** | ***p*-value**^*^ | **% (95% CI)** | ***p*-value**^*^ | **% (95% CI)** | ***p*-value**^*^ |
| **Total population** |  |  |  |  |  |  |
| Migraine, No.; HCs, No. | 630; 153 | - | 630; 148 | - | - | - |
| Migraine vs HCs | 37.3 (11.5 to 69.2) | **0.003** | 30.4 (8.8 to 56.3) | **0.004** | - | - |
| **History of aura** |  |  |  |  |  |  |
| Migraine, No.; HCs, No. | 630; 153 | - | 630; 148 | - | 630;0 | - |
| MA vs MO | 21.5 (-5.0 to 55.4) | 0.17 | 16.2 (-5.9 to 43.4) | 0.26 | 14.7 (-3.8 to 36.8) | 0.13 |
| MA vs HCs | 57.6 (15.8 to 114.3) | **0.001** | 31.0 (9.9 to 47.2) | **0.003** | - | - |
| MO vs HCs | 29.7 (-0.4 to 68.9) | 0.056 | 19.9 (-0.7 to 36.3) | 0.062 | - | - |
| **Diagnosis of chronic migraine** |  |  |  |  |  |  |
| Migraine, No.; HCs, No. | 630;153 | - | 630; 148 | - | 630;0 | - |
| CM vs EM | 19.9 (-5.3 to 51.7) | 0.20 | 7.1 (-12.6 to 31.1) | > 0.99 | 4.0 (-13.3 to 24.7) | 0.68 |
| CM vs HCs | 46.4 (12.0 to 91.2) | **0.002** | 33.5 (5.8 to 68.5) | **0.009** | - | - |
| EM vs HCs | 22.1 (-9.2 to 64.3) | 0.32 | 24.7 (-3.6 to 61.4) | 0.12 | - | - |
| **Headache status at blood sampling** |  |  |  |  |  |  |
| Migraine, No.; HCs, No. | 510;153 | - | 510; 148 | - | 510;0 | - |
| Ictal vs Interictal | -2.2 (-24.2 to 26.2) | > 0.99 | -5.7 (-24.2 to 17.3) | > 0.99 | -11.2 (-26.8 to 7.6) | 0.23 |
| Ictal vs HCs | 40.3 (5.3 to 86.8) | **0.014** | 29.4 (0.9 to 65.9) | **0.039** | - | - |
| Interictal vs HCs | 43.4 (6.3 to 93.4) | **0.012** | 37.2 (5.7 to 78.0) | **0.011** | - | - |
| **Abbreviations:** BMI, body mass index; CI, confidence interval; CM, chronic migraine; EM, episodic migraine; HCs, healthy controls; IQR, interquartile range; MA, migraine with aura; MO, migraine without aura; SD, standard deviation. **Symbols:** *, adjusted for multiple testing using the Bonferroni method; -, not applicable. | | | | | | |

eTable 7. Estimated relative differences in serum hs-CRP (sensitivity analysis; exclusion of hs-CRP values >10.0 mg/L)

|  | **Estimated relative difference** | | | | | |
| --- | --- | --- | --- | --- | --- | --- |
|  | **Unadjusted model** | | **Adjusted for age, sex, BMI, and smoking** | | **Fully adjusted model** | |
|  | **% (95% CI)** | ***p*-value**^*^ | **% (95% CI)** | ***p*-value**^*^ | **% (95% CI)** | ***p*-value**^*^ |
| **Total population** |  |  |  |  |  |  |
| Migraine, No.; HCs, No. | 610; 149 | - | 601; 144 | - | - | - |
| Migraine vs HCs | 36.3 (12.2 to 65.6) | **0.002** | 30.3 (9.7 to 54.8) | **0.003** | - | - |
| **History of aura** |  |  |  |  |  |  |
| Migraine, No.; HCs, No. | 610; 149 | - | 601; 144 | - | 543;0 | - |
| MA vs MO | 12.2 (-11.1 to 41.7) | 0.70 | 11.7 (-8.8 to 36.9) | 0.57 | 14.8 (-4.1 to 37.4) | 0.13 |
| MA vs HCs | 48.0 (10.7 to 97.8) | **0.004** | 41.1 (9.2 to 82.4) | **0.004** | - | - |
| MO vs HCs | 31.8 (3.0 to 68.9) | **0.022** | 26.3 (1.6 to 57.0) | **0.031** | - | - |
| **Diagnosis of chronic migraine** |  |  |  |  |  |  |
| Migraine, No.; HCs, No. | 610; 149 | - | 601; 144 | - | 543;0 | - |
| CM vs EM | 18.4 (-5.0 to 47.7) | 0.20 | 7.0 (-11.8 to 29.8) | > 0.99 | 1.7 (-15.4 to 22.3) | 0.85 |
| CM vs HCs | 44.7 (12.7 to 85.8) | **0.001** | 33.4 (7.0 to 66.4) | **0.005** | - | - |
| EM vs HCs | 22.2 (-7.4 to 61.2) | 0.25 | 24.7 (-2.4 to 59.3) | 0.093 | - | - |
| **Headache status at blood sampling** |  |  |  |  |  |  |
| Migraine, No.; HCs, No. | 493;149 | - | 487;144 | - | 445;0 | - |
| Ictal vs Interictal | -3.0 (-23.7 to 23.3) | > 0.99 | -1.7 (-20.2 to 21.1) | > 0.99 | -10.8 (-26.5 to 8.4) | 0.25 |
| Ictal vs HCs | 38.1 (5.5 to 80.8) | **0.012** | 31.9 (4.0 to 67.2) | **0.016** | - | - |
| Interictal vs HCs | 42.4 (7.5 to 88.6) | **0.008** | 34.2 (4.6 to 72.0) | **0.014** | - | - |
| **Abbreviations:** BMI, body mass index; CI, confidence interval; CM, chronic migraine; EM, episodic migraine; HCs, healthy controls; IQR, interquartile range; MA, migraine with aura; MO, migraine without aura; SD, standard deviation. **Symbols:** *, adjusted for multiple testing using the Bonferroni method; -, not applicable. | | | | | | |
